# P53 and The Immune Response: 40 Years of Exploration—A Plan for the Future

**DOI:** 10.3390/ijms21020541

**Published:** 2020-01-15

**Authors:** Arnold J. Levine

**Affiliations:** Simons Center for Systems Biology, Institute for Advanced, Study, Princeton, NJ 08540, USA; alevine@ias.edu

**Keywords:** Tp53 and p53, cancer biology, immunology, the innate immune response, the adaptive immune response, life span, senescence, epigenetics and development

## Abstract

The *p53* field was born from a marriage of the techniques of cancer virus research and immunology. Over the past 40 years, it has followed the path of cancer research. Now cancer treatments are turning to immunotherapy, and there are many hints of the role of the p53 protein in both the regulation of the innate immune system and as an antigen in adaptive immune responses. The *p53* gene and protein are part of the innate immune system, and play an important role in infectious diseases, senescence, aging, and the surveillance of repetitive DNA and RNAs. The mutant form of the p53 protein in cancers elicits both a B-cell antibody response (a tumor antigen) and a CD-8 killer T-cell response (a tumor-specific transplantation antigen). The future will take the *p53*-immune response field of research into cancer immunotherapy, autoimmunity, inflammatory responses, neuro-degeneration, aging, and life span, and the regulation of epigenetic stability and tissue regeneration. The next 40 years will bring the *p53* gene and its proteins out of a cancer focus and into an organismic and environmental focus.

## 1. Introduction

Forty years ago, four research groups in London, Paris, New York City/Bethesda, MD, and Princeton, NJ, USA, discovered the p53 protein in a variety of cancerous cells and normal cells employing antisera from animals carrying SV40 virus-induced tumors or spontaneously-derived tumors [1,2,3,4]. These observations began a set of studies on the role of *p53* in cancers that has led to the conclusion that *p53* mutations are the most common feature of human cancers. Over 40 years of research publications and 17 meetings dedicated to the p53 gene and its protein, cancer biology has been the subject under discussion. But it could have happened another way, focusing instead upon role of *p53* in the immune response and immunology, with research largely being carried out by immunologists. Lloyd Olds was a noted tumor immunologist, David Lane had just completed a PhD in the laboratory of an immunologist, and Arnold Levine taught immunology courses at Princeton University, so the authors of the first papers might have pursued the role of the p53 protein in adaptive and innate immunity, and not only in cancer biology. This article celebrates forty years of *p53* research and pays tribute to the possibility that, in the future, immunologists will be attracted to *p53* research efforts. In that spirit, this review will explore the idea that p53 is part of the innate immune system, and that one day, p53 cancer antigens will be targets of immunotherapy. After all, if 50% of human cancers have p53 mutations, and if the immune system recognizes foreign tumor antigens targeting them for cellular rejection, these two lines of research will make a good marriage.

## 2. Tumor Antigens and the Adaptive Immune System

During the 1950s and 1960s, a number of DNA viruses that were able to cause tumors in rodents and humans were isolated, e.g., SV40, polyoma, several of the human adenoviruses, some human papilloma viruses, and human Epstein Barr virus [5]. In every case, the viral genome or portions of the viral genome persisted in the tumor cells, being either integrated into a cellular chromosome or as a plasmid. For each of these viruses, a portion of that viral genome was expressed as m-RNAs, which were translated into viral proteins [6]. In all of these cases, except for the Epstein-Barr virus, these viral proteins were demonstrated to be involved in the initiation and maintenance of tumor formation [5,6,7,8,9]. Because these viral proteins were not encoded for in the host genome, the immune system recognized them as foreign and produced antibodies directed against them. These antibodies became primary tools to detect viral oncoproteins, which were classified as tumor antigens (TA). In some cases, animals immunized with these virally-induced tumors were able to reject a subsequent inoculation of tumor tissue. The antigens mediating tumor rejection were tumor antigens that were termed “tumor-specific transplantation antigens” (TSTA) [10]. The adaptive immune response composed of macrophages and dendritic cells presenting an antigen, CD-4 helper Th-2 T-cells, and the B-cells producing IgG antibodies (TA) and CD-4 helpers Th-1 T-cells that signal to CD-8 killer T-cells (TSTA), responded to these viral encoded antigens. Thus, the adaptive immune response was very active against these viral encoded antigens.

In addition to these viral antigens expressed in tumors, several fetal antigens, such as alpha fetoprotein (liver cancer) and the carcino-embryonic antigen (colon cancer) were expressed only in actively replicating fetal cells, and not in adult tissue, but were reexpressed in the cancerous tissue. These antigens also elicited antibodies directed against the fetal antigens produced in the tumor.

With this background, in 1979, four different research groups probing quite different sets of questions uncovered the existence of the p53 protein, employing antisera from tumor-bearing animals [1,2,3,4]. DeLeo and his colleagues immunized and inoculated isogenic mice with a spontaneously-transformed and tumorigenic cell line, and employed the antisera from these mice to detect the p53 protein in extracts of the tumorigenic cell line. Kress employed the sera from SV40 tumor-bearing animals to detect both the SV40 large T-antigen and a second protein, p53. Lane and Crawford detected both the SV40 T antigen and p53, and found that upon dilution of the antisera, the ratio of T-antigen and the p53 protein remained constant, suggesting that these two proteins formed a complex in solution and were co-immuno-precipitated together. Linzer and Levine inoculated hamsters with the SV40 virus and induced tumors in these animals. The sera were employed to detect the p53 protein in SV40-infected and -transformed mouse and monkey cell extracts. The same p53 protein (based upon peptide maps) was found in SV40-transformed mouse cells and murine cancer cells not produced by a virus. The p53 protein was detected at much lower levels in uninfected normal cells. The results in these four publications suggested that the SV40 tumor antigen, which was known to be responsible for the initiation and maintenance of transformation and tumor formation, bound to cellular protein p53 and increased its concentration compared with levels in normal cells. Cancer cells not produced by any virus also expressed high levels of the p53 protein, whereas normal cells had much lower levels. In addition, the cellular protein p53 became immunogenic in cancer cells, and so CD-4 Th2 T-cells had receptors for the protein and acted as helpers to permit p53-specific antibody production by B-cells.

Linzer and his colleagues [11] then demonstrated that temperature-sensitive mutations in the SV40 gene that encoded the T-antigen regulated the levels of the p53 protein in virus-infected and -transformed cells in a temperature-sensitive fashion. At the permissive temperature, the cells were transformed and T-antigen p53 complexes were formed with high levels of the p53 protein. At nonpermissive temperatures, where the SV40 T-antigen does not function, the cells were normal and p53 levels were very low. Crawford and his colleagues showed that about 10% of women with breast cancers produced antibodies against the human p53 protein [12]. Datasets of human antibodies directed against the p53 protein from people with a variety of cancers demonstrated that about 30% of individuals with cancers have p53 protein detectable antibodies [13]. These antibodies appear to be quite specific to p53, and are associated with cancers that have p53 missense mutations. When the epitopes on the p53 protein where the p53 antibodies bound were mapped, the antibodies tended to react with the N- and C-termini of p53 protein. This is not where the missense mutations in the p53 protein are located. About 50% of human cancers have p53 mutations, and most of those are missense mutations that map to the DNA-binding domain of the p53 protein. Many of these mutant p53 proteins fail to bind to DNA and no longer function as a transcription factor. The p53 transcription factor transcribes the MDM-2 gene, which produces a protein, i.e., the E3 ubiquitin ligase of the p53 protein. This produces an autoregulatory loop where the p53 protein promotes the transcription of the MDM-2 gene, which, in turn, produces the MDM-2 protein that polyubiquitinates the p53 protein and helps to degrade it [14]. Thus, p53 missense mutations fail to transcribe the MDM-2 gene and to degrade the mutant p53 protein. As a result, mutant p53 proteins are at high levels in transformed or cancerous cells, and at much lower levels in normal cells with a wild-type p53 gene. This may help to explain why only mutant p53 proteins result in antibody production. Peptides produced from these proteins are at high levels and have the affinity to bind to Class-2 (HLA) and CD-4 T cell receptors (TCRs), helping B-cells make IgG antibodies. The epitopes for these antibodies do not have to recognize the missense mutation; they recognize the higher concentrations of p53 peptides from the N- and C-terminal ends of the protein. Raising the levels of a cellular protein (self) in a cell can trigger an immune response.

Clearly, there is a good adaptive B-cell response to a mutant p53 protein. Is there a CD-8 killer T-cell response directed against the p53 antigen? Balachandran and his colleagues [15] have explored the CD-8 T cell responses in human cancer patients with pancreatic cancers. They employed peptide antigens derived from mutated oncogenes and tumor suppressor genes (neoantigens) presented by class 1 HLA receptors in cancer cells or associated macrophages produced by individuals who were long-term survivors (10 years or more) of pancreatic cancers. Normally, about 93–95% of patients diagnosed with pancreatic cancer live less than five years. About 3–7% of these patients are alive at ten years or more after diagnosis. In this study, patients who had survived pancreatic cancer for ten years or more were studied. These patients were treated with surgery and chemotherapy. No immunotherapy was employed. The DNA from their original tumors was sequenced and the oncogene and tumor suppressor gene mutations were identified. The CD-8 T-cell receptor sequences were also obtained from tissue sections of the original tumors. Knowing the HLA types of the class 1 receptors from each patient made it possible to estimate the off-rate constant of a peptide from its class 1 receptor using the NETMHC algorithm [15,16]. This was multiplied by the degree of homology of the peptide sequence to a known antigen derived from the microbiome, as listed in the immune epitope database (IEDB) [15,16]. The idea behind this was that a prior exposure of the immune system to a cross-reacting antigen provided by an infectious disease would prime the immune response to a tumor antigen. This calculation produced presumptive neoantigens that were derived from mutated oncogenes and tumor suppressor genes in the pancreatic cancers. Most pancreatic cancers have both RAS mutations and p53 mutations. Ten years after diagnosis, seven patients had their blood drawn to obtain peripheral blood leukocytes. The monocytes and dendritic cells were allowed to attach to a solid surface. They were incubated with nine amino acid-length peptides that were found in the mutant proteins of oncogenes and tumor suppressor genes calculated to bind well (i.e., a NETMHC off constant of below 500nM) to the class 1 receptor on those cells. These target cells were then incubated with CD-8 T cells to determine whether these T-cells would recognize the antigen presented. The CD-8 T-cells killing the target cells then replicate in response to engaging an antigen. All of these cells had their T-cell receptors sequenced at the start of the experiment, day zero, and 21 days after the incubation of the T-cells with the target cells. The number of T-cell receptor sequence DNA reads gives the number of cells, and therefore, the degree of replication of a T-cell clone after 21 days of incubation. The most common antigen found to stimulate the CD-8 T-cells from long-term survivors was muc16, a cellular encoded protein that is neither an oncogene nor a tumor suppressor gene, but which might protect the cells from an immune response. Interestingly, one patient, a 10.5 year survivor, had a CD-8 T cell clone in her blood ten years after diagnosis that was directed against the peptide that had the p53 missense mutation, E191K (an E to K amino acid change at codon 271). The sequence of the T-cell receptor that bound to this p53 mutant peptide could also be found in the paraffin-embedded tissue section of the original tumor ten years earlier, and was at a frequency of 0.11% of the T-cells detected in that original pancreatic tumor. The p53 mutant peptide was homologous to epitopes found in the IEDB, making it a good candidate as a tumor specific neoantigen or a TSTA [17] that cross reacts with an infectious agent. P53 mutant peptides were also reported to be good neoantigens for both CD-4 and CD-8 T-cell receptors from cancer patients by Rosenberg and his colleagues [18].

Rather clearly, the mutant p53 protein is recognized by the adaptive immune response in patients with cancers. It might one day be found to play a role in checkpoint therapy or even in CAR-T cell therapy. Selected p53 mutations, with the right HLA type, could exert enough immune responses to keep a tumor under control and permit a patient to become a long-term survivor. One might even begin to carefully test mutant p53 peptides as vaccines for selected cancers with the right HLA type and a specific p53 mutation. We might be concerned about an autoimmune response against the wild-type p53 protein; low levels of the wild-type p53 protein in normal cells might avoid such an autoimmune reaction.

This will become a new area for p53 researchers over the next few years. In these examples, the recognition of the p53 epitopes by the class 2 receptors, CD-4 helper, and B-cells (at the N- and C-terminal peptides) is clearly different from the recognition of the p53 epitope (at the mutational site) by the class 1 receptors, CD-4 TH-1, and CD-8 killer T cell receptors (TCR). It is not surprising that the two arms of the adaptive immune system react with distinct p53 protein epitopes with very different affinity constants (antibodies commonly have nano-molar affinities, whereas CD-8 TCR’s have micro-molar affinities).

## 3. The Innate Immune Response, Infectious Diseases, and p53

P53 was discovered because it formed a complex with the SV40 tumor antigen [2,3,4]. The retinoblastoma protein (Rb) also forms a protein complex with the SV40 tumor antigen [19], and in both cases, the viral oncoprotein T-antigen inactivates the functions of each of the tumor suppressor genes. The SV40 T-antigen inactivates the Rb protein to move the virus-infected cell from the G-0 or G-1 phase of the cell cycle into the S-phase, so as to have the cell synthesize all the precursors and substrates for optimal viral (and cellular) DNA replication. When the T-antigen moves a cell into the S-phase without stimulation by growth factors and the synthesis of cyclins, that should be observed as a stress by p53, which would then be activated as a transcription factor, and p53 would kill the virus-infected cell, limiting viral replication. The virus responds by inactivating p53 before it can kill the cell. All of the small DNA tumor viruses (SV40, adenoviruses [20], and papilloma viruses [8,9] contain viral tumor antigens that inactivate Rb and p53 [21]. Interestingly the p53 protein is targeted for inactivation by many viruses that form tumors, or even those that do not. Both DNA and RNA virus-infected cells signal a number of different stress signals that activate p53-mediated transcription, resulting in the killing of cells unless the virus successfully inactivates the p53 protein functions. Because of this, almost every successful virus has developed a way to inactivate p53. This means that the p53 gene and protein are part of the cellular defenses termed “innate immune responses”. In addition, there are bacteria that target p53 in cells for inactivation. *Helicobacter pylori* attaches to gastric epithelial cells of the stomach and inserts its Cag A protein into the cell, which results in the activation of the AKT protein kinase. This protein kinase phosphorylates the MDM-2 protein and activates it, enhancing its E3 ligase activity and inactivating p53 [22]. *H. pylori* is known to be associated with gastric cancers. The role of p53 in preventing viral and bacterial infections and diseases, and the role of infectious agents in countering p53 activity, open up new avenues of research. For example, the drug nutlin is known to block the MDM-2 protein from binding to p53 and inactivating it. Could nutlin become an antibiotic for *H. pylori* inactivation of p53? Yes, the treatment of *H. pylori*-infected gastric cells with nutlin liberates p53 from inhibition and kills the bacterial-infected cells [23]. Thus, nutlin could become a novel antibiotic. The role of the innate immune response and p53 in protecting the host from infectious diseases will be an important area of the p53 research of the future.

## 4. Cellular Senescence, p53, the Innate Immune Response, and Aging

Among the more remarkable stress signals that activate the wild type p53 transcription factor are the mutations in selected oncogenes such as Myc, Ras, or Ets. In tumors that contain high levels of Ras mutations such as nonsmall cell lung cancers (NSCLC), colon cancers, or pancreatic cancers, p53 mutations are almost always also observed in the same tumor cells. When the Ras oncogene is employed to transfect normal rat embryo fibroblasts with a wild type p53 gene, the cells in the culture commonly die of senescence [24]. If the p53 gene and protein are mutated and lose their function, the cells live and are transformed. An activated mutant K-Ras pathway is a stress that is sensed by the p53 protein and the cell is placed into senescence, which is commonly an irreversible loss of cell division. The K-Ras pathway activates the transcription of the Ets gene, which promotes the transcription of the tumor suppressor ARF. The ARF protein binds to the MDM-2 E-3 ubiquitin ligase, which, in turn, inactivates this ligase, increasing the half-life of the p53 protein. The increased levels of the p53 protein turn on a transcriptional pathway, which includes the p21 protein, PAI-1, PML, and mir-34a, which stop the cell cycle and promote the changes leading to senescence. When this happens in vivo, the senescent cells secrete a number of cytokines and interleukins (TNF, IL-6, etc.) which attract NK cells, CD-8 killer T-cells, and monocytes/macrophages, which kill the senescent cell and clean up the cellular debris. This is termed the “senescence-associated secretory phenotype”, or SASP [25]. In this case, the activation of the p53 transcription factor in the presence of mutant K-Ras is part of the innate immune system whose job it is to eliminate senescent cells from the body. If senescent cells persist in the body and are not killed, the cytokines such as IL-6 and TNF cause a number of diseases associated with aging, such as rheumatoid arthritis, inflammatory diseases, autoimmune disorders, and even cancers.

As we age, the efficiency of the immune system declines. It has been postulated that the persistence of senescent cells drives the aging process [26]. Kirkland and colleagues have demonstrated that adding senescent cells to young mice causes persistent physical dysfunction [27]. Adding senescent cells to older mice resulted in a reduced survival of that cohort compared to age match untreated mice. The senolytic drugs desatinib plus quercetin decrease the number of naturally-occurring senescent cells and reduce the SASP in older mice, decreasing the physical dysfunction of these mice and extending post-treatment survival or life-span by 36%. Human adipose cell explants with senescent cells producing a SASP were treated with this senolytic combination, which then reduced the number of senescent cells and the levels of the SASP. These experiments support the idea that aging and its associated frailties and disorders could well be brought on by the continual formation of senescent cells secreting cytokines (a p53 activated SASP) which are not removed by the NK cells, CD-8 T-cells, and macrophages and dendritic cells. These ideas are consistent with an important role of the innate immune system and cells under the control of p53 in natural aging and frailty. Several other publications have also implicated the p53/MDM-2 axis with aging or premature aging [28,29] Thus, p53 responding to a variety of time-dependent stresses could bring on aging processes mediated by senescence, SASP, and the innate immune response, resulting in cytokines that give rise to diseases and frailty.

## 5. Repetitive DNA Sequences in the Genome, p53, and Associated Immune Responses

Slightly more than 50% of the DNA sequences in the human genome are composed of repetitive DNAs [30]. A number of these sequence elements come from retroviruses and retrotransposons that have colonized the genome over evolutionary time frames. These sequences are largely kept transcriptionally silent over most of the postfetal life span of the person by epigenetic control and by being part of the heterochromatin. It has become clear that the p53 protein plays a role in silencing these repetitive sequences. [30,31] In cancer cells with p53 mutations, many of these diverse repetitive elements are expressed [30]. Haber, Ting, and colleagues isolated cancer cells from blood samples of patients and sequenced the RNAs of these cells. In some cases, heterogeneous repetitive RNA sequences, not observed in normal cells, compose up to 30% of the total RNA in these cancer cells [30,31,32]. Many of these RNA species are retrotransposons (Line elements, HERVs, HSAT-2) [30], and of particular interest is the observation of a repetitive element called “HSAT-2” [30,31,32,33]. HSAT-2 has an abnormally high CpG content for the human genome. The DNA of this retrotransposon resides in the centromere region of several human chromosomes. In cancer cells, this DNA is transcriptionally expressed. The RNA produced is not translated, but instead, copied by reverse transcriptase (possibly derived from Line elements or HERV’s), and the DNA formed integrates back into the genome at or near the centromere of some human chromosomes. Thus, with time, the copy number increases and the HSAT-2 RNA levels become very high in cancer cells. These high CpG content RNAs get packaged into exosomes and are shipped out of the cancer cells where they are then phagocytized by macrophages and dendritic cells. In macrophages and dendritic cells, the RNAs induce interferon, IL-6, and TNF from the innate immune system [34]. Thus, in any situation, like cancer, that results in the expression of high CpG repetitive DNA elements, these RNAs can trigger an innate immune response. The wild-type and mutant *p53* molecules play a role in all of this by silencing the transcription of repetitive elements (wild-type *p53*), or after a mutation, permitting the transcription of repetitive elements. *P53* is not the only check upon silencing repetitive elements in a cell [30], but it is an important one in cancer cells. In this case, even the absence of the wild-type p53 protein (from a deletion mutation) results in an innate immune response [33].

Interestingly, the very same set of events happens in some virus-infected cells when the virus inactivates p53 functions. [34]. In Cytomegalovirus and Herpes simplex types 1- and 2-infected cells, HSAT-2 is induced to transcribe its CpG rich sequences after infection. This requires some viral functions that inactivate *p53* function. The production of HSAT-2 RNA and DNA is essential for obtaining optimal virus titers, but the reasons for this remain obscure. However, in vivo, CMV and HSV-1 and -2 infected cell cytoplasms produce exosomes, which are taken up by macrophages. For CMV, this is the cell type in which it becomes latent and remains in the body over a lifetime. For HSV-1 and -2, this should result in antigen presentation to adaptive immune responsive cells, and ultimate clearance of the virus until reactivation [34].

## 6. Conclusions

An immune system is essential to all life forms. Every organism alive today has developed methods to identify and reject foreign DNA or RNA. Eubacteria and Archaea have developed various Crispr systems, while invertebrates employ macrophage-like cells to eliminate bacteria, and have an innate immune system. Vertebrates, starting with fish, employ both innate and adaptive immune systems. A p53-like protein was first found in early multicellular animals like the Choanoflagellates [35]. Invertebrates localized the p53 like functions in their germ line and the p53 protein was first focused upon by detecting DNA damage repair and recombination [36]. Although mutations and natural selection generate diversity in a species, the p53 protein maintains fidelity. This included a role for p53 in the detection, elimination, or silencing of repetitive DNA, and retrotransposons. As the innate immune system developed, it is likely that the p53-like protein in invertebrates included innate immune responses as part of its signal transduction pathway, responding to stresses and infectious diseases. With time, detection and responses to metabolic stresses were added to the p53 pathway of worms [36]. With further evolutionary time, the development of vertebrate tissue repair, regeneration, and tissue-specific stem cells gave rise to cancerous growth, and the Tp53 gene and its protein were repurposed to the somatic tissues where they responded to cancerous growths [30]. In this evolutionary fashion, the mammalian p53 protein forms a node, i.e., the MDM-2-p53 node, that in the totality of the cells’ signal transduction pathways is the most highly-connected node of this cellular pathway. As such, it can be calculated to have the highest information content or highest entropy [37]. Among its functional connections are the detection of foreign genomes or RNA, and the repression of repetitive elements (the p53 protein binds to half sites of DNA sequences in LINES, HERVS, and many retrotransposons) [38]. In parallel, the innate immune system detects and responds to foreign DNA and RNA and foreign patterns of lipids, carbohydrates, and proteins. The adaptive immune system detects and responds to foreign antigens. The appearance of foreign DNA, RNA, and proteins in and by the organism is seen as a stress. It is this common enemy that unites the p53 response with the immune response.

Many examples of this have been discussed in this review. The wild-type p53 protein is part of the innate immune response. Even when the p53 protein contains a mutation and is not functional, the transcription of repetitive DNAs in the cell triggers an innate immune response. Thus, the p53 mutant protein has a function even as it loses its transcription factor activity, in its failure to bind to DNA and repress transcription. By virtue of its autoregulatory cycle with MDM-2, the mutant p53 protein increases its concentration in a cell, and the peptides from a degraded mutant p53 are presented by macrophages and class 2 receptors to B-cells and CD-4 Th-2 helper T-cells to activate an antibody response and mature that response to an IgG class antibody. A class 2 receptor encoded by the Killer/Dr5 gene is a p53 transcriptionally regulated gene [39]. Binding to class 1 HLA receptors, the CD-4 Th-1 TCRs and the CD-8 killer T-cell TCRs detect a different set of peptides from the p53 mutant protein (i.e., a neoantigen with the p53 mutation) than antibodies (i.e., the N- and C-terminal peptides of the p53 protein).

This review is meant to lay the groundwork for the p53 field to attract immunologists who will flesh out the circuitry between the p53 pathway and the immune system. Some of this is interactive and reinforcing, and some aspects of the p53 stress system and the NFK-beta immune system are antagonistic [40]. High levels of p53 turn down NFK-beta activity, and high levels of NFK-beta activity decrease p53 functioning. This, in part, comes from the fact that the immune system amplifies its products by clonal cellular replication, and p53 shuts down cellular replication when it detects a stress. Yet, p53 needs NFK-beta to employ innate and adaptive responses. We do not yet understand how the interactions of these two transcription factors are regulated, nor do we understand how these two systems can, in times of stress, call forth a truce and cooperate. Antibiotics do not really function without the immune system, and cancer chemotherapy is likely not successful without the immune system. The answers to these questions are intimately involved in the study of infectious diseases, autoimmunity, cancer immunotherapy, repetitive DNA and RNA elements, and a deeper understanding of the circuitries of the p53 pathway and the immune system. It is time to begin these studies.
